# Inhibiting the Recruitment of PLCγ1 to Kaposi’s Sarcoma Herpesvirus K15 Protein Reduces the Invasiveness and Angiogenesis of Infected Endothelial Cells

**DOI:** 10.1371/journal.ppat.1005105

**Published:** 2015-08-21

**Authors:** Silvia Gramolelli, Magdalena Weidner-Glunde, Bizunesh Abere, Abel Viejo-Borbolla, Kiran Bala, Jessica Rückert, Elisabeth Kremmer, Thomas F. Schulz

**Affiliations:** 1 Institute of Virology, Hannover Medical School, Hannover, Germany; 2 German Center for Infection Research (DZIF), Braunschweig, Germany; 3 Institute of Molecular Immunology, Helmholtz Center Munich, German Research Center for Environmental Health (GmbH), Munich, Germany; University of Pennsylvania Medical School, UNITED STATES

## Abstract

Kaposi’s sarcoma (KS), caused by Kaposi’s sarcoma herpesvirus (KSHV), is a highly vascularised tumour of endothelial origin. KSHV infected endothelial cells show increased invasiveness and angiogenesis. Here, we report that the KSHV K15 protein, which we showed previously to contribute to KSHV-induced angiogenesis, is also involved in KSHV-mediated invasiveness in a PLCγ1-dependent manner. We identified βPIX, GIT1 and cdc42, downstream effectors of PLCγ1 in cell migration, as K15 interacting partners and as contributors to KSHV-triggered invasiveness. We mapped the interaction between PLCγ1, PLCγ2 and their individual domains with two K15 alleles, P and M. We found that the PLCγ2 cSH2 domain, by binding to K15P, can be used as dominant negative inhibitor of the K15P-PLCγ1 interaction, K15P-dependent PLCγ1 phosphorylation, NFAT-dependent promoter activation and the increased invasiveness and angiogenic properties of KSHV infected endothelial cells. We increased the binding of the PLCγ2 cSH2 domain for K15P by substituting two amino acids, thereby creating an improved dominant negative inhibitor of the K15P-dependent PLCγ1 activation. Taken together, these results demonstrate a necessary role of K15 in the increased invasiveness and angiogenesis of KSHV infected endothelial cells and suggest the K15-PLCγ1 interaction as a possible new target for inhibiting the angiogenic and invasive properties of KSHV.

## Author Summary

Kaposi’s Sarcoma (KS), etiologically linked to Kaposi’s sarcoma herpesvirus (KSHV), is a tumour of endothelial origin characterised by angiogenesis and invasiveness. *In vitro*, KSHV infected endothelial cells display an increased invasiveness and high angiogenicity. Here we report that the KSHV protein K15, which increases the angiogenicity of endothelial cells, contributes to KSHV-mediated invasiveness by the recruitment and activation of the cellular protein PLCγ1 and its downstream effectors βPIX, GIT1 and cdc42. We explored the functional consequences of disrupting the K15-PLCγ1 interaction by using an isolated PLCγ2 cSH2 domain as a dominant negative inhibitor. This protein fragment, by interacting with K15, reduces K15-driven recruitment and activation of PLCγ1 in a dose-dependent manner. Moreover, the PCLγ2 cSH2 domain, when overexpressed in KSHV infected endothelial cells, reduces the angiogenesis and invasiveness induced by the virus. These findings highlight the role of the K15-PLCγ1 interaction in KSHV-mediated invasiveness and identify it as a possible therapeutic target.

## Introduction

Kaposi’s Sarcoma Herpesvirus (KSHV), also known as human herpesvirus 8 (HHV-8), was first identified in Kaposi’s sarcoma (KS) tissues by representational difference analysis [1,2]. Found to be the etiological agent of KS, the virus was successively linked also to primary effusion lymphoma (PEL) and multicentric Castleman’s disease (MCD) [3,4], two rare lymphoproliferative disorders.

KS, a highly vascularized tumour, is one of the most frequent AIDS-related cancers and a major health problem in sub-Saharan Africa [5,6]. Histologically KS is characterised by the presence of an inflammatory infiltrate of neutrophils, B and plasma cells as well as aberrant angiogenesis [7,8]. In advanced KS lesions, the main proliferative elements are the KSHV infected endothelial cells, which lose their typical morphology, become spindle-shaped and acquire invasive properties [9]. Although the majority of KSHV infected endothelial cells in KS tissue harbour the virus in a latent phase, a small population undergoes lytic (productive) reactivation [10]. Moreover, lytically infected cells secrete viral and cellular factors able to promote the pathological angiogenesis and invasiveness of latently infected cells in a paracrine manner [11–13]. In particular, upon lytic reactivation, virus infected endothelial cells show an increased secretion of VEGF, Ang2, ephrin B2, MMPs, IL6 and 8 [12,14,15]. Among the KSHV-encoded proteins that possess angiogenic and invasive properties are the latency associated nuclear antigen 1 (LANA1), the viral homolog of G-protein coupled receptor (vGPCR, a constitutively active IL8 receptor), the viral homolog of interleukin 6 (vIL6), two viral chemokine homologs, vCCL1 and 2, as well as the K1 protein [12,14–16]. In this context, we recently reported that the KSHV K15 protein also contributes to KSHV-mediated angiogenesis [17].

**Fig 1:**
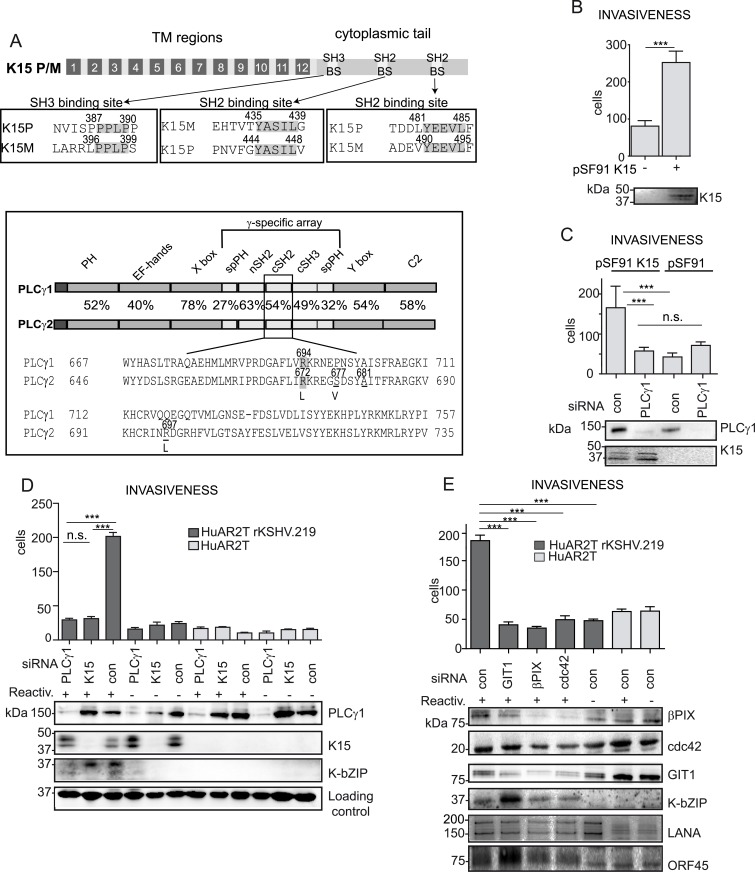
K15 confers invasiveness to KSHV infected endothelial cells in a PLCγ1-dependent manner. (A) upper panel: schematic diagram of K15; alignments of the K15P and M SH3 and SH2 binding sites (BS) are boxed. Bottom panel: PLCγ1 and PLCγ2 domain architecture, the percentages indicate the sequence conservation between the two proteins. A sequence alignment for the cSH2 domain is shown, highlighted are the conserved arginine, underlined are the residues mutated to generate the PLCγ2 cSH2 superbinder (SB) mutant. (B) K15 overexpression increases the number of invasive cells. Invasion assay in HuAR2T transduced with a retroviral vector for K15 (+) or the control vector (-). K15 expression was confirmed by western blot. For representative images see S1A Fig. (C) Invasion assay as in (B) in the presence of the indicated siRNAs. For representative images, see S1B Fig. (D) Silencing the expression of PLCγ1 and K15 reduces the invasiveness of KSHV infected endothelial cells. Invasion assay in KSHV infected HuAR2T transfected with the indicated siRNAs, forty-eight hours after the induction of the lytic cycle (indicated with +). Protein expression levels were evaluated by western blot. For representative images see S1C Fig. (E) GIT1, βPIX and cdc42 contribute to KSHV mediated invasiveness. Invasion assay as in (D), in the presence of the indicated siRNAs. For representative images see S1D Fig. ***, p< 0.001; n.s., non significant.

The K15 gene, located at the “right end” of the long unique region (LUR) of the viral genome, consists of eight exons that are alternatively spliced [18–20]. Two main K15 alleles, termed P (predominant) and M (minor) have been identified with the M allele most likely being the result of a homologous recombination with an unknown γ2 herpesvirus [21]. For both K15P and M a mRNA comprising all eight exons is the most strongly expressed transcript and encodes a protein of 12 predicted transmembrane domains and a cytoplasmic tail involved in signalling. Although K15P and M share as little as 33% of amino acid sequence homology, the cytoplasmic tail of both K15 alleles contains a putative src homology -3 (SH3) (PLPP) and two SH2 (YASIL, YEEVL) binding sites (Fig 1A, upper panel) [18–20]. Previous studies reported that both K15 alleles activate NF-κB and the Ras/MAPK signalling [22–25]. Microarray studies revealed that K15 upregulates the expression of genes involved in angiogenesis and cell migration [22,24,25], including, among others, COX2 and an NFAT-dependent upregulation of DSCR1 [17,24,25]. We previously showed that K15 contributes to the increased angiogenic properties of KSHV infected primary endothelial cells in a matrigel-based capillary tube formation assay [17]. This process involves the K15P-dependent recruitment of phospholipase C γ1 (PLCγ1) and its phosphorylation on tyrosine 783, the successive activation of the calcineurin/NFAT pathway and the downstream upregulation of DSCR1 [17].

Apart from its role in angiogenenesis, PLCγ1 is also required for cell migration and cytoskeleton remodelling in both cancer and normal endothelial cells [26–30]. Upon integrin engagement, PLCγ1 associates with the βPIX (β-PAK interacting/exchange factor)-GIT1 (G-protein-coupled receptor kinase-interacting protein 1) complex leading to the activation of the small GTPase cdc42 and subsequent cell spreading and migration [27]. Furthermore, in cancer patients, PLCγ1 overexpression and increased phosphorylation levels are correlated with poor prognosis and metastasis formation [31,32]. Activating somatic mutations in PLCγ1 have recently been observed in several cases of angiosarcoma [33–35]. In addition to the ubiquitously expressed PLCγ1, the PLCγ protein family also includes PLCγ2 which is found mainly in hematopoietic cells. These two isoforms share the same domain organization and a high degree of sequence homology (Fig 1A, bottom panel). Both are characterized by the presence of the γ-specific array, a protein region of approximately 500 amino acids inserted between the two modules of the catalytic domain (Fig 1A, bottom panel). The γ-specific array consists of a split pleckstrin homology domain (spPH), two SH2 as well as a SH3 domain, and is required for the regulation of PLCγ enzymatic activity.

In this study, we report that the recruitment and activation of PLCγ1 by K15 contributes to the invasiveness of KSHV infected endothelial cells in a GIT1- βPIX- and cdc42-dependent manner. We also show that the overexpression of an isolated SH2 domain, derived from PLCγ2, can disrupt the K15P-PLCγ1 interaction and can inhibit K15-dependent downstream signalling and thereby the increased invasiveness and angiogenic properties of KSHV infected endothelial cells.

## Results

### K15 and PLCγ1 contribute to the invasiveness of KSHV infected endothelial cells

Previously reported microarray experiments have shown that K15 increases the expression of genes involved in inflammation and cell migration such as chemokines and MMPs [17,25]. Moreover, we and others [17,36,37] had suggested that K15 expression increased the invasiveness of endothelial cells. This is illustrated in Fig 1B and S1A Fig, which compare the invasiveness of K15-transduced immortalized endothelial cells (HuAR2T) to that of cells transduced with the control vector. In this experimental system, K15 overexpression significantly increases the invasiveness of endothelial cells. Since we previously reported that K15 recruits and activates PLCγ1 [17], which has been found to trigger cellular invasiveness in many cellular contexts [28,32], we tested whether the recruitment of PLCγ1 by K15 was also important for K15-driven invasiveness. We performed invasion assays using K15-transduced endothelial cells (HuAR2T) transfected with either control siRNA or siRNA targeting PLCγ1 (Fig 1C and S1B Fig). Silencing PLCγ1 was sufficient to decrease K15-mediated invasiveness to the basal level, indicating that K15 increases the invasiveness of endothelial cells in a PLCγ1-dependent manner. We and others have previously shown that, upon lytic reactivation, KSHV infected endothelial cells display an increased ability to invade the extracellular matrix [12,14,16]. To elucidate the contribution of K15 and PLCγ1 to the KSHV-mediated invasiveness induring the induction of the lytic cycle, KSHV infected immortalized endothelial cells (HuAR2T rKSHV.219) were transfected with siRNA targeting either K15 or PLCγ1 and the invasiveness of the cells was evaluated in a matrigel-based invasion assay (Fig 1D and S1C Fig). Upon induction of the lytic cycle, the cells showed increased invasiveness (Fig 1D, compare lane 3 to lane 6). Depletion of either K15 or PLCγ1 by siRNA significantly reduced the number of invading cells (Fig 1D, compare lanes 1 and 2 to lane 3). Furthermore, reduced invasiveness was also observed in HEK 293 stably harbouring a recombinant KSHV genome lacking K15 (KSHV BAC36 ΔK15) as compared to cells harbouring KSHV wt genome (KSHV BAC36) (S2 Fig). These results point to K15 and PLCγ1 as contributors to KSHV-mediated invasiveness (Fig 1D and S1C and S2 Figs).

Since it has been reported that βPIX, GIT1 and cdc42 act downstream of PLCγ1 and contribute to integrin-mediated invasiveness [27,28], we silenced these proteins (using siRNA) and tested the invasiveness of KSHV infected endothelial cells, following the reactivation of the lytic cycle. Although the siRNA pools used in this experiment did not completely suppress the expression of βPIX, GIT1 and cdc42, they significantly reduced the invasiveness of KSHV infected endothelial cells, compared to the control siRNA treated cells (Fig 1E and S1D Fig). Therefore, we conclude that K15 contributes to KSHV-induced invasiveness and that this process is mediated, at least in part, by PLCγ1 and its downstream effectors GIT1, βPIX and cdc42.

### K15 recruits GIT1, PLCγ1 and cdc42 in KSHV infected endothelial cells

**Fig 2:**
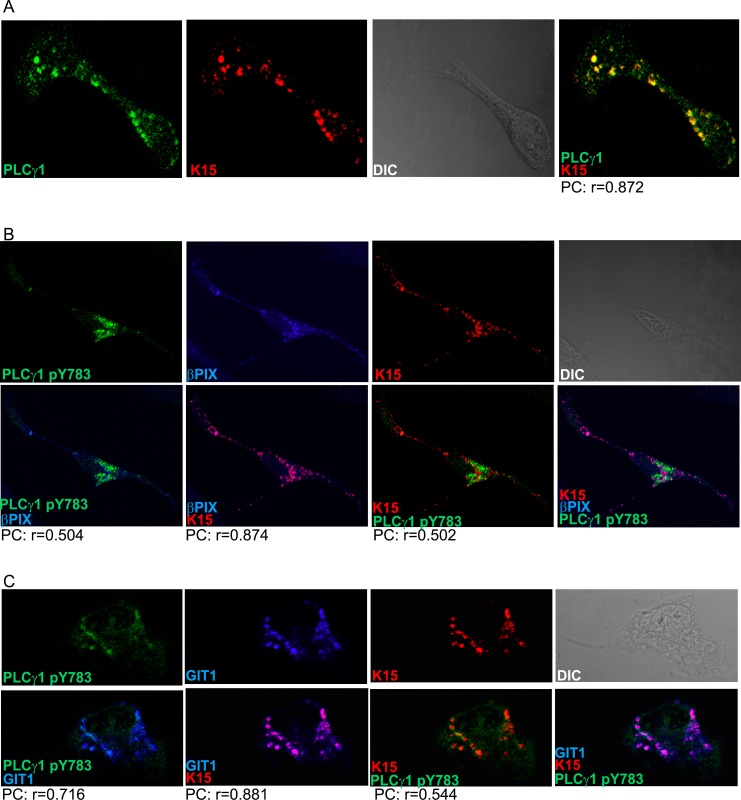
K15, PLCγ1, βPIX and GIT1 co-localize in latent KSHV infected endothelial cells. Immunofluorescence assay in HuAR2TrKSHV.219 cells. (A) Co-staining of K15 (red) and PLCγ1 (green). (B) and (C) Co-staining of K15 (red), phosphorylated (active) PLCγ1 (green) and βPIX or GIT1 (blue). The co-localization of the three proteins is visible in the merge image as white colour. The co-localisation was analysed using the JACOP tool and the Pearson’s coefficient (PC) was calculated.

**Fig 3:**
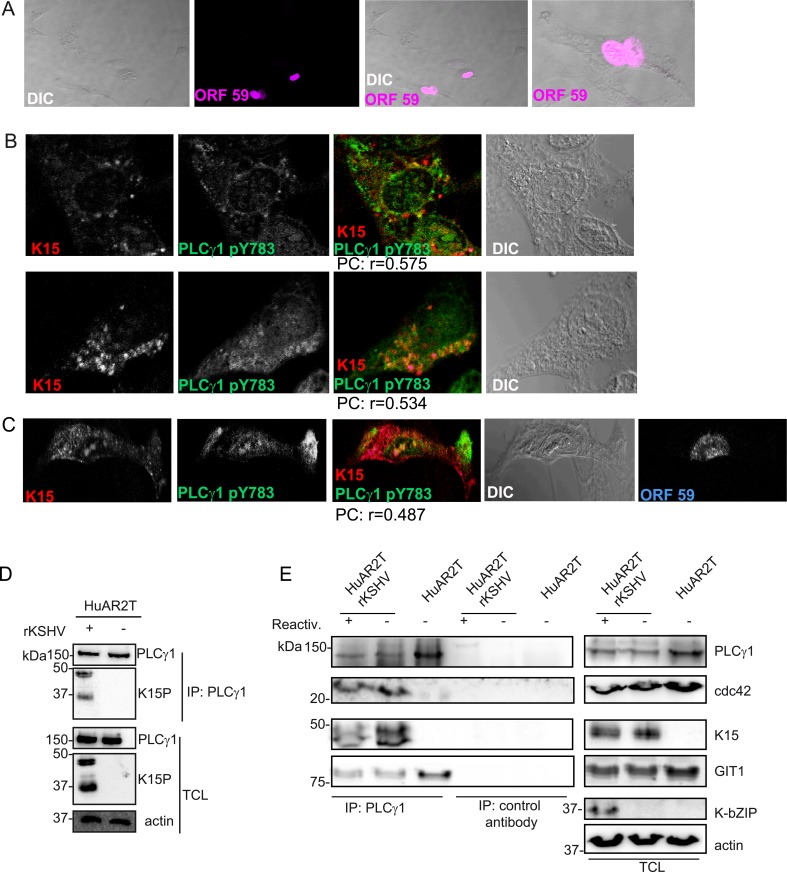
K15 interacts with PLCγ1, GIT1 and cdc42 in KSHV infected endothelial cells. (A) HuAR2TrKSHV cells expressing ORF59, stained with an antibody to orf59 and a cy3-labelled second antibody and shown in pseudo-color purple, seventy two hours following activation of the lytic cycle. (B) Co-localisation of cy5 labelled K15 (pseudocolor red), FITC labelled phosphorylated (active) PLCγ1 (green) in ORF59-negative HuAR2TrKSHV cells seventy-two hours after reactivation of the lytic cycle. (C) Co-localisation of cy5 labelled K15, FITC labelled phosphorylated (active) PLCγ1 in ORF59-positive HuAR2TrKSHV cells. (D) Co-immunoprecipitation of K15 with PLCγ1 in HuAR2TrKSHV.219 cells. (E) Co-immunoprecipitation using either an antibody to PLCγ1 or a control antibody (rabbit anti-Flag) with K15, cdc42 and GIT1 in HuAR2T and HuAR2T rKSHV following induction (+) or not (-) of the lytic cycle. TCL: total cell lysate.

We next investigated the cellular localization of K15, PLCγ1, βPIX and GIT1 in KSHV infected cells by immunofluorescence assay. K15 staining has previously been performed in HEK293T or HeLa cells in the context of transient transfection [22,25]. Under these experimental conditions, K15 was localised at the plasma membrane showing a punctate pattern or in large patches with a perinuclear distribution. A similar distribution has also been reported in HEK 293 cells stably transfected with KSHV BAC 36 [25]. Using a newly generated rat monoclonal antibody to K15 (see Materials and Methods), we were able to detect endogenously expressed K15 in KSHV infected endothelial cells (Fig 2). In the cultures shown here, about 15–20% of KSHV infected cells were expressing K15 to a detectable level. The majority of K15-expressing cells showed a perinuclear as well as a plasma membrane localization with a punctate pattern, with bigger dots localizing in the perinuclear region (Fig 2A–2C and Fig 3A), in line with a previous report from our group in which a polyclonal antibody to K15 was used [25]. Interestingly, in these dot-like structures, K15 protein co-localized strongly with total PLCγ1 and partially with phosphorylated PLCγ1 (pY783 PLCγ1), as well as with βPIX and GIT1 (Fig 2A–2C).

In order to address the question if K15 co-localises with activated (phosphorylated) PLCγ1 in infected cultures undergoing the early stages of lytic replication, we co-stained KSHV infected HuAR2T cells, in which the lytic replication cycle had been induced, with antibodies to K15 and PLCγ1 phosphorylated on Y783. We confirmed the induction of the lytic cycle by staining the early KSHV protein encoded by orf 59 (polymerase processivity factor) (Fig 3A). Similarly to what we observed in latently infected HuAR2T (Fig 2A–2C), in lytically induced cultures, we also observed partial co-localisation of phosphorylated PLCγ1 and K15 (Fig 3B). In these cultures, this co-localisation was mainly observed in orf59-negative cells (Fig 3B) and it was less evident in orf 59 positive cells (Fig 3C). Owing to the lack of antibodies to βPIX and GIT1 suitable for co-localisation studies with K15 in cultures stained for orf59, we could not assess whether, as in the case of phosphorylated PLCγ1, βPIX and GIT1 co-localize with K15 in HuAR2T-KSHV cell cultures after the activation of the lytic cycle.

We also investigated the interaction between K15, PLCγ1, cdc42 and GIT1 by co-immunoprecipitation. As shown in Fig 3D and 3E, we co-immunoprecipitated PLCγ1 from KSHV infected and uninfected endothelial cells (HuAR2TrKSHV.219 and HuAR2T) and detected its interaction with K15 in the former, but not the latter cell line. We also observed the previously reported interaction of PLCγ1 with GIT1 in infected and uninfected cells (Fig 3E). Moreover, we observed that, in KSHV infected but not in uninfected cells, PLCγ1 associates not only with K15 and GIT1, but also with cdc42 (Fig 3E). Furthermore, when we immunoprecipitated PLCγ1 from KSHV infected endothelial cells following reactivation of the lytic cycle (Fig 3E), we also observed a complex composed of K15, PLCγ1, GIT and cdc 42 (Fig 3E).

Thus, K15 associates with PLCγ1, GIT1 and cdc42 in cells of both latently and lytically induced cultures. Based on the immunofluorescence experiment shown in Fig 3A–3C, we conclude that the association of K15 with PLCγ1, GIT1 and cdc42 occurs mainly in latent cells (i.e. cells lacking orf59 expression). Unfortunately, low levels of βPIX expression in combination with the lack of an antibody suitable for western blot did not allow us to assess the presence of βPIX in this complex by co-immunoprecipitation.

Since it has been previously shown that GIT1 forms a stable complex with βPIX and that these proteins serve as a scaffold for the assembly of pro-migratory cytoplasmic protein complexes induced by activated PLCγ1 [38], K15 might recruit these proteins to trigger virus-induced cellular invasiveness.

### The PLCγ1 cSH2 domain contributes to the binding to K15

**Fig 4:**
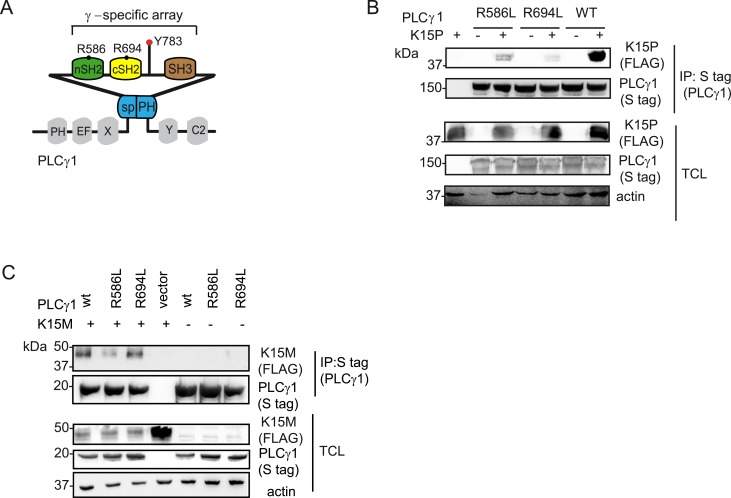
PLCγ1 SH2 domains are important for the binding to K15P and M. (A) Schematic diagram of and PLCγ 1, the γ-specific array motifs are highlighted in colour and the amino acid position for the conserved arginine residues within the c and nSH2 domains are indicated. (B) and (C) Co-immunoprecipitation assay of K15P (B) and K15M (C) by S-tagged PLCγ1 wt, or its mutants R586L, R694L in HEK293T cells.

Previously, we showed that the C-terminal SH2 binding motif (YEEV) and SH3 binding motif (PPLP) of K15P (Fig 1A) both contribute to the interaction with PLCγ1 [17]. In this study we wanted to identify the PLCγ1 region involved in this interaction. Since the γ-specific array region of PLCγ1contains two SH2 domains (nSH2 and cSH2) (Fig 1A, bottom panel, Fig 4A and [39–41]), we focused on this portion of PLCγ1. The SH2 domain is a protein module of approximately 100 amino acids with an evolutionarily conserved phospho-tyrosine binding site. In particular, an arginine within the binding pocket is highly conserved in human SH2 domains and provides nearly half of the ligand binding energy [42,43]. To investigate the potential role of the two PLCγ1 SH2 domains in the interaction with K15, we mutated the conserved arginine residues within both the n and c SH2 domains (R586L and R694L, respectively; see Figs 1A and 4A) and tested these PLCγ1mutants for binding to both the K15P and M variants, after co-transfection with K15 expressing vectors into HEK293T cells. While mutation of the nSH2 domain (R586L) moderately decreased the binding to K15P, mutation of the cSH2 domain (R694L) had a stronger effect on this interaction (Fig 4B). In the case of K15M, only the mutant of the nSH2 domain of PLCγ1 (R586L) showed a reduced binding (Fig 4C). These results indicate that between K15P and K15M there is a subtle difference with regard to how they interact with PLCγ1. While the N-terminal SH2 domain of PLCγ1 seems to be involved in the interaction with both K15P and K15M, the cSH2 domain is only important for the binding to K15P.

### K15 binds to the isolated cSH2 domain of PLCγ2

**Fig 5:**
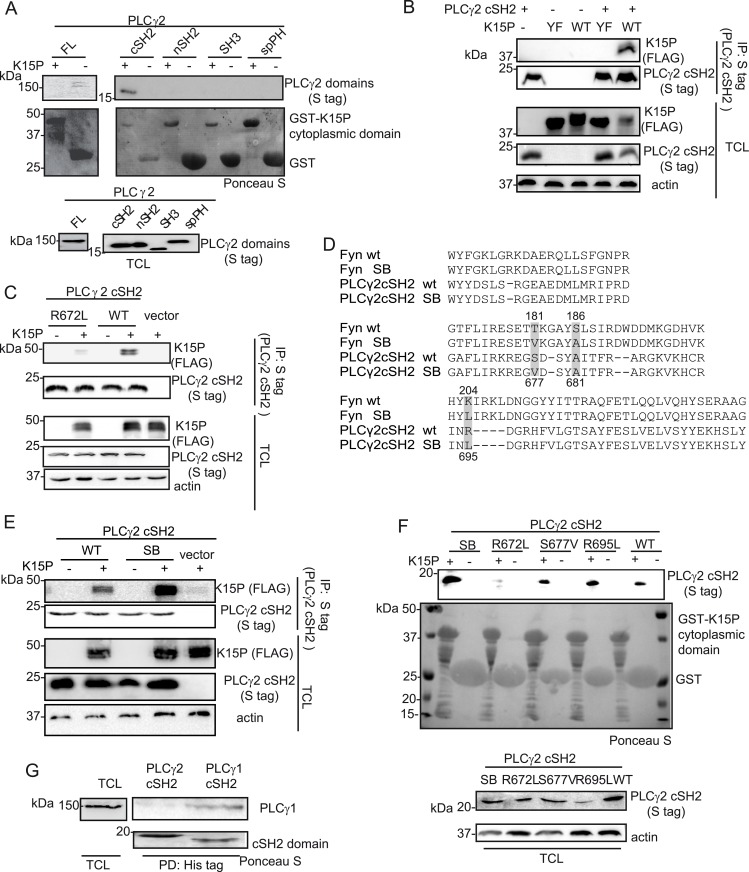
The isolated PLCγ2 cSH2 domain binds to K15. (A) The isolated PLCγ2 cSH2 domain, but no other PLCγ2 domain, binds to the K15P cytoplasmic domain. GST-pulldown assay with GST-K15P cytoplasmic domain and either full length (FL) PLCγ2 (left) or individual PLCγ2 domains (right) expressed in HEK293T cells (B) A mutation within the YEEV SH2 binding motif of K15 disrupts the interaction with the PLCγ2 cSH2 domain. Co-immunoprecipitation assay with S tag PLCγ2 cSH2 domain and K15P wt or Y481F (YF) mutant in HEK293T cells. (C) Mutation of the conserved R672 within the isolated PLCγ2 cSH2 domain impairs binding to K15. Co-immunoprecipitation assay with S tag PLCγ2 cSH2 wt and R672L mutant domain and K15P wt in HEK293T. (D) Alignment of the protein sequence of the SH2 domains of Fyn and PLCγ2; amino acids mutated to generate the SB SH2 domains of Fyn and PLCγ2 are highlighted. Numbering above the sequences refers to the Fyn sequence, below to the PLCγ2 sequence. SB: superbinder (see text) (E) The PLCγ2 cSH2 SB shows an increased affinity for K15. Co-immunoprecipitation assay with PLCγ2 cSH2 wt and SB domain and K15P in HEK293T. (F) GST-pulldown assay with GST-K15P cytoplasmic domain and wt or mutant PLCγ2 cSH2 domain. (G) The isolated cSH2 domain of PLCγ1, but not of PLCγ2 binds to PLCγ1. Pulldown of His-cSH2 isolated domains of PLCγ1 and 2 and endogenous PLCγ1 from HEK293T cellular lysate.

In metazoans, there are two isoforms of PLCγ, the ubiquitously expressed PLCγ1, and PLCγ2, whose expression is restricted mainly to hematopoietic cells. Although they are functionally distinct, the two isoforms share some binding partners. We therefore explored the possibility that K15 might bind to PLCγ2 as well. To this end, we performed a GST pulldown assay with a fusion protein of GST and the K15P cytoplasmic domain (aa 347–489) bound to glutathione beads and a lysate of HEK293T cells that had been transiently transfected with plasmid expressing PLCγ2 or individual PLCγ2 domains. Although we could not observe an interaction between the K15P cytoplasmic domain and the full length PLCγ2 protein (Fig 5A, left panel), the PLCγ2 cSH2 domain, in isolation, did bind to K15P (Fig 5A, right panel).

Since the interaction between K15 and PLCγ1 is required for both KSHV mediated angiogenesis [17] and invasiveness (Fig 1D), we wondered if a targeted inhibition of this interaction could have therapeutic potential. PLCγ1 is ubiquitously expressed and depletion of the PLCγ1 gene in mice leads to embryonic lethality [44], hence its inhibition would be expected to be associated with side effects in uninfected cells. However, an inhibitor that would specifically target the interaction between K15 and PLCγ1, without interfering with the physiological functions of PLCγ1 might counteract only KSHV-dependent signalling processes. Since Decker et al [45] successfully impaired pathological osteolysis by uncoupling the adaptor and the catalytical functions of PLCγ2 by ectopic expression of tandem PLCγ2 SH2 domains, we used a similar approach and tested the PLCγ2 cSH2 domain as a potential inhibitor of the K15-PLCγ1 interaction. We first characterized the interaction between K15P and the isolated PLCγ2 cSH2 domain in more detail. To confirm that the interaction between K15 and the PLCγ2 cSH2 domain takes place through the YEEV SH2 binding site of K15, which has been shown to participate in the interaction with PLCγ1 [17], we mutated the conserved tyrosine in this motif to phenylalanine (K15 Y481F, from now on called K15 YF). Fig 5B shows that the K15P YF mutant does not associate with the PLCγ2 cSH2 domain. We also mutated the conserved arginine (R672L) (Fig 1A, bottom panel) within the PLCγ2 cSH2 domain. Fig 5C shows that this mutation impairs binding to K15P. Therefore, the interaction between the PLCγ2 cSH2 and K15P involves the Y481 of K15 and R672 of the PLCγ2 cSH2 domain.

### A superbinder PLCγ2 SH2 domain binds more efficiently to K15

It has been previously reported [46] that the phospho-tyrosine binding pocket of the SH2 domain of the cellular kinase Fyn can be optimized in order to obtain stronger binding to a phosphorylated binding site, without changing the binding specificity of this SH2 domain. The authors obtained this superbinder (SB) SH2 domain by mutating specific amino acids (T181V; S186A; K204L) within the Fyn SH2 domain. As both sequence and structure of SH2 domains are highly conserved, we identified and mutated the corresponding amino acids in the isolated PLCγ2 cSH2 domain (Fig 5D) in order to obtain a SB mutant. Of the residues altered to obtain the Fyn SB SH2 domains (Fig 5D), one, the A681, was already an alanine in the PLCγ2 cSH2 domain. We therefore mutated only two residues, S677 and R695 to valine and leucine, respectively. Subsequently, we tested the ability of K15P to associate with the PLCγ2 cSH2 S677V/R695L mutant (from now on called superbinder, SB) by co-immunoprecipitation (Fig 5E). K15 was precipitated more efficiently with the SB mutant than with the wt cSH2 domain (Fig 5E). This result was confirmed by GST pulldown from lysates of cells transfected with expression vectors for the PLCγ2 cSH2 wt or SB domain (Fig 5F). The K15P cytoplasmic tail fused to GST bound more efficiently to the SB than to either of the single mutants (S677V and R695L) of the cSH2 domain (Fig 5F). This result is in line with the observations reported by Kaneko et al. [46] that the properties of the SB mutant are the result of a synergistic effect obtained by mutating multiple residues in the SH2 domain. To confirm that the cytoplasmic domain of K15 binds directly to the PLCγ2 cSH2 domain or its SB mutant, we used surface plasmon resonance (SPR) to measure the interaction of purified, prokaryotically expressed GST-K15P and the PLCγ2 cSH2 wt and SB domains, as described in Materials and Methods. We obtained equilibrium dissociation constants (K_D_) of 5.8 10^−9^ for PLCγ2 cSH2 wt, and 3.8 10^−9^ for the SB mutant This result shows that the interaction of K15 with the PLCγ2 cSH2 domain is of high affinity, in the nanomolar range, even though in this experiment K15, having been expressed in bacteria, was not phosphorylated on Y489. This lack of phosphorylation most likely explains the similar affinity observed for the interaction of K15 with the PLCγ2 cSH2 wt and SB domains.

Since the cSH2 domain of PLCγ1 has been shown to contact the phosphorylated Y783 in PLCγ1 [47,48], thereby mediating an intramolecular interaction required for the activation of this protein, we also investigated if the isolated PLCγ2 cSH2 would interact with PLCγ1. Purified His-tag fused PLCγ2 cSH2 domain bound to Ni-beads was incubated with cleared cell lysate and was probed *via* western blot for its binding to endogenous PLCγ1. The PLCγ1 cSH2 domain was included in the experiment as a positive control. Endogenous PLCγ1 was pulled down by the isolated PLCγ1 cSH2 domain, but not by the PLCγ2 cSH2 domain (Fig 5G). Thus, the PLCγ2 cSH2 isolated domain interacts with K15 (*via* its YEEV motif, Fig 5B), but not with PLCγ1.

### The PLCγ2 cSH2 domain impairs the interaction of K15P with PLCγ1 as well as K15 mediated downstream signalling

**Fig 6:**
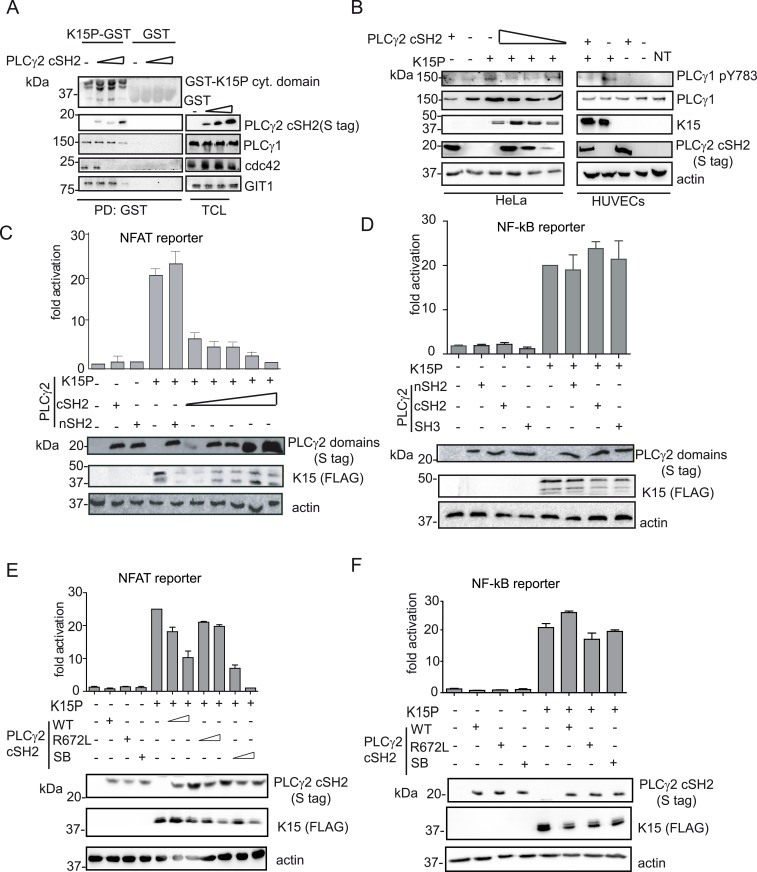
The isolated PLCγ2 cSH2 domain affects K15-mediated signalling in a dominant negative manner. (A) Increasing amounts of the isolated PLCγ2 cSH2 domain gradually inhibit the interaction of K15 with PLCγ1, GIT1 and cdc42. The GST-K15P cytoplasmic domain was used to pulldown endogenous PLCγ1, GIT1 and cdc42 from lysates of HEK293T transfected with 0.5, 1 or 2 μg of a PLCγ2 cSH2 domain expressing plasmid. Top panel: Ponceau S staining; remaining panels: western blot with indicated antibodies. (B) Increasing amounts of the isolated PLCγ2 cSH2 domain decrease K15P-induced PLCγ1 phosphorylation. Western blot analysis of lysates from HeLa CNX cells co-transfected with K15 expressing construct and increasing amounts (0.5, 1, 2 μg) of vector encoding the PLCγ2 cSH2 domain (left panel) or primary HUVECs transduced with a retrovirus expressing K15 and a lentivirus expressing PLCγ2 cSH2 (right panel). (C)-(F) Luciferase reporter assay. (C) Increasing amounts of the PLCγ2 cSH2 domain decrease K15-induced NFAT activation in a dose-dependent manner. Activation of NFAT-dependent promoter in presence of K15P and either increasing amounts (0.5, 1, 2 μg) of the PLCγ2 cSH2 or the nSH2 domain (2μg) in HEK293T. (D) None of the PLCγ2 isolated domains interfere with K15 induced NF-κB activation. Activation of NF-κB-responsive promoter in the presence of K15 and different PLCγ2 domains in HeLa CNX cells. (E); (F) Activation of the indicated promoter in HEK293T cells (E) or in HeLa CNX cells (F) in the presence of K15 and increasing amounts (1, 2 μg) of wt, SB or binding deficient (R672L) PLCγ2 cSH2 domain.

It was previously reported that the overexpression of the PLCγ1 γ-specific array in cancer cell lines has a dominant negative effect on PLCγ-meditated cell migration [29,30]. Moreover, as shown above, K15 co-localizes with PLCγ1, GIT1, βPIX and these proteins contribute to KSHV-mediated invasiveness (Figs 1, 2 and 3). Since we also observed that the PLCγ2 cSH2 domain interacts with K15 *via* its YEEV motif, which is required for downstream signalling (Fig 5B), we decided to test if it has a dominant negative effect on the interaction of K15 with PLCγ1 and the subsequent signalling events. In an interaction assay, increasing amounts of the PLCγ2 cSH2 domain gradually reduced the binding of PLCγ1, cdc42 and GIT1 to the GST-K15 cytoplasmic tail (Fig 6A). In this assay, higher concentrations of the isolated PLCγ2 cSH2 domain were needed to disrupt the binding of PLCγ1 and GIT1 to K15 than to reduce the binding of cdc42. In addition, the presence of the isolated PLCγ2 cSH2 domain reduced in a dose-dependent manner K15-mediated PLCγ1 phosphorylation levels in HeLa cells, as well as in primary HUVECs (Fig 6B).

We have previously shown that K15 induces angiogenesis by binding and activating PLCγ1 thereby triggering NFAT-mediated signalling [17]. Therefore, we also investigated the effect of the PLCγ2 cSH2 domain on K15-mediated activation of the NFAT-dependent promoter in a luciferase based reporter assay (Fig 6C). Increasing amounts of the PLCγ2 cSH2 domain gradually decreased the ability of K15 to activate an NFAT-dependent promoter, further confirming that the PLCγ2 cSH2 domain could be used as a dominant negative inhibitor of K15-mediated signalling. In contrast, the nSH2 domain of PLCγ2, which does not bind to K15 (Fig 5A), also did not inhibit the K15-dependent NFAT activation.

K15 mediates the activation of several cellular signalling cascades, including the NF-κB pathway [22,23,25]. We previously showed that the activation of NF-κB by K15 occurs *via* a region in the K15 cytoplasmic tail located close to the last transmembrane domain [23]. To explore the specificity of the dominant negative effect observed with the PLCγ2 cSH2 domain, we tested whether it would also affect the K15-mediated activation of NF-κB (Fig 6D). Neither the isolated PLCγ2 cSH2 domain, nor the isolated PLCγ2 nSH2 and SH3 domains (which do not bind K15, Fig 5A) compromised the ability of K15 to activate NF-κB-dependent transcription. Furthermore, we tested the effect of the SB (S677V/R695L) and the R672L mutants of the PLCγ2 cSH2 domain on the K15-mediated NFAT- as well as NF-κB activation (Fig 6E and 6F). While the SB showed a stronger effect than the PLCγ2 cSH2 wt, the R672L mutant had no effect on the ability of K15 to activate NFAT-dependent transcription (Fig 6E). In contrast, neither the SB nor the R672L mutant had any effect on the ability of K15 to activate an NF-κB-dependent promoter (Fig 6F). Therefore, we conclude that the PLCγ2 cSH2 domain specifically impairs K15-mediated NFAT activation, but has no effect on NF-κB-dependent transcription. In addition, increasing the affinity of the PLCγ2 cSH2 domain for the K15 YEEV motif, by introducing two substitutions (Fig 5D), enhances its dominant negative effect on K15-dependent NFAT activation, but has no influence on NF-κB signalling.

### The PLCγ2 cSH2 domain has no effect on K15M-dependent signalling

**Fig 7:**
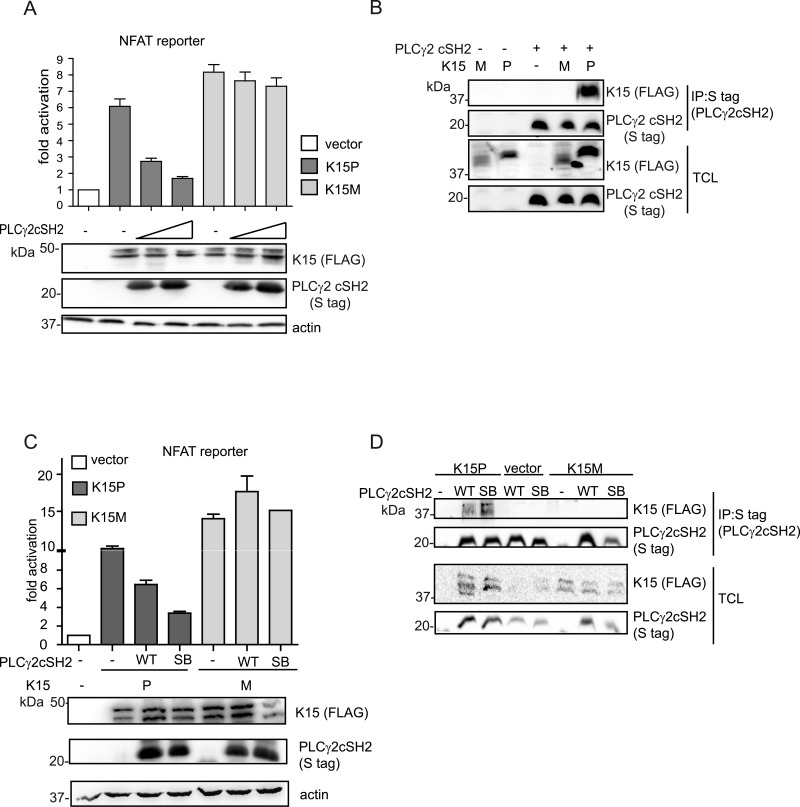
The isolated PLCγ2 cSH2 domain does not interact with K15M and has no effect on the activation of an NFAT promoter by K15M. (A) Increasing amounts of the PLCγ2 cSH2 domain have no effect on K15M-induced NFAT activation. Activation of NFAT reporter in the presence of K15P and M expressing vectors and two different amounts (0.5, 1.5 μg) of constructs expressing the PLCγ2 cSH2 domain transfected into HEK293T cells. (B) The PLCγ2 cSH2 domain binds to K15P, but not to K15M. Co-immunoprecipitation assay with S tagged PLCγ2 cSH2 domain wt and K15P or M in HEK293T cells. (C) The PLCγ2 cSH2 SB does not affect the activation of an NFAT-dependent promoter by K15M. Activation of NFAT reporter by K15P and M in the presence of wt or SB PLCγ2 cSH2 domain (1.5 μg of transfected DNA) (D) The PLCγ2 cSH2 SB does not bind to K15M. Co-immunoprecipitation assay with S tagged PLCγ2 wt or SB cSH2 domain and K15P or M.

As described above, the other K15 allele, K15M, also binds to PLCγ1 *via* the PLCγ1 nSH2 domain but differs subtly from K15P, whose binding to PLCγ1 appears to involve additionally the PLCγ1 cSH2 domain (Fig 4C). Similar to K15P, K15M can also activate NFAT-dependent promoters [24]. Therefore we investigated the effect of the PLCγ2 cSH2 domain on K15M-mediated NFAT-dependent transcriptional activation (Fig 7A). Irrespective of the amount of transfected PLCγ2 cSH2 plasmid, we did not detect any significant difference in K15M-mediated NFAT-dependent activation, although in the same assay a dose dependent inhibitory effect was observed for K15P. Consequently, we decided to test the ability of K15M to bind the isolated PLCγ2 cSH2 domain but could not detect any association between these two proteins (Fig 7B). Furthermore, we tested whether the expression of the PLCγ2 cSH2 SB could affect K15M-triggered activation of an NFAT-dependent promoter. To this end, we compared the ability of both K15M and P to activate NFAT signalling in the presence of the wt or SB PLCγ2 cSH2 domain. While the presence of both wt and SB PLCγ2 cSH2 domain reduced K15P-dependent NFAT activation, neither of these two proteins affected the ability of K15M to activate the NFAT-dependent promoter (Fig 7C). To explore whether the PLCγ2 cSH2 domain SB would interact with K15M, we also tested the binding of the wt and the SB cSH2 domain to K15M in a co-immunoprecipitation assay (Fig 7D). While the PLCγ2 cSH2 SB domain, as expected, showed increased binding to K15P, it failed, as did the wt cSH2 domain, to interact with K15M (Fig 7D). Therefore, while the PLCγ2 cSH2 domain binds K15P and thereby blocks K15P-dependent NFAT signalling, it does not bind K15M and, consequently, has no influence on the K15M-mediated activation of NFAT-dependent transcription. Mutations that increase the ability of the PLCγ2 cSH2 domain to interact with K15P do not confer binding to K15M.

### The PLCγ2 cSH2 domain impairs KSHV-mediated invasiveness and angiogenesis

**Fig 8:**
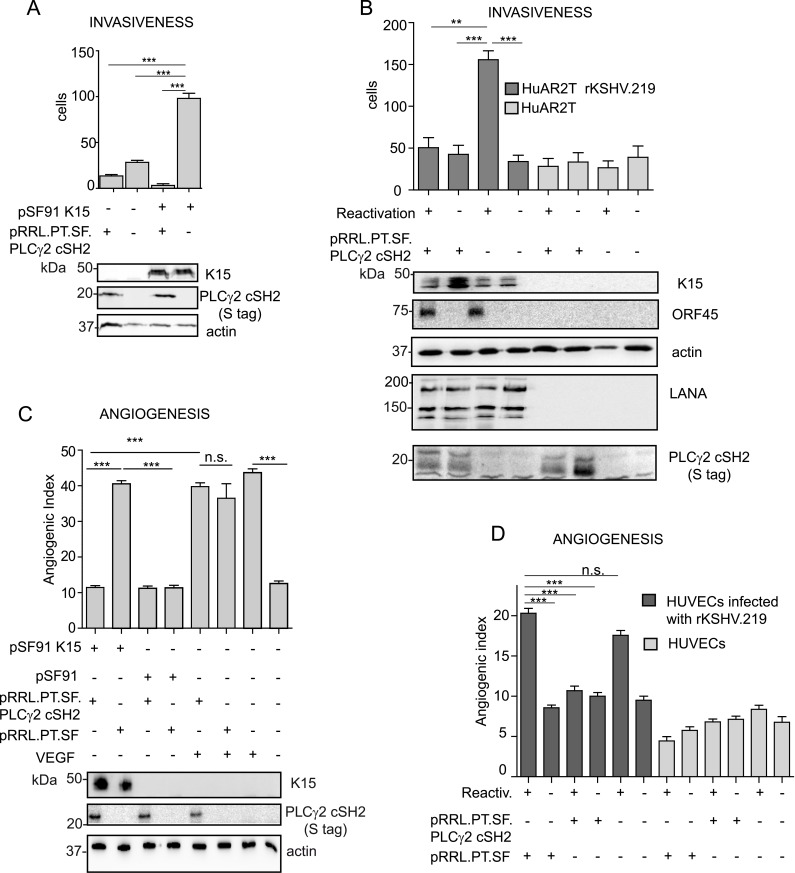
The isolated PLCγ2 cSH2 domain impairs the increased invasiveness and angiogenesis of KSHV infected endothelial cells. (A) The PLCγ2 cSH2 domain reduces K15P-mediated invasiveness. Invasion assay in HuAR2T transduced with a retroviral vector for K15P (pSF91 K15) and a lentiviral vector for the PLCγ2 cSH2 domain (pRRL.PT.SF.PLCγ2 cSH2). (-) indicates cultures transduced with the corresponding control retro- (pSF91) or lentiviral (pRRL.pT.SF) vectors. Expression of K15 and PLCγ2 cSH2 was confirmed by western blot. For representative images see S3A Fig. (B) The PLCγ2 cSH2 domain reduces the increased invasiveness of KSHV infected endothelial cells. Invasion assay in HuAR2T rKSHV.219 cells treated as indicated. (-) indicates no activation of the lytic cycle (top row) or transduction with the control lentiviral vector pRRL.pT.SF. Protein expression levels were detected by western blot. The immediate-early KSHV protein orf45 was used as a marker for the lytic cycle and KSHV LANA as a marker for infected cells. For representative images see S3B Fig. (C) The PLCγ2 cSH2 domain reduces K15-mediated angiogenesis. Angiogenic tube formation in HUVECs transduced with the indicated retroviral or lentiviral vectors or treated with 50 ng/ml of VEGF. Expression of K15 and the PLCγ2 cSH2 domain was confirmed by western blot. For representative images see S3C Fig. (D) The PLCγ2 cSH2 domain reduces KSHV-mediated angiogenesis. Angiogenic tube formation in HUVECs infected with rKSHV.219, transduced with a lentivirus expressing the PLCγ2 cSH2 domain or the control vector; where indicated (+) the lytic cycle was induced. For representative images see S3D Fig. ***, p< 0.001; n.s., non significant.

So far, our results showed that the isolated PLCγ2 cSH2 domain inhibits K15-mediated NFAT activation by blocking the recruitment of PLCγ1. We further wanted to test the effect of the PLCγ2 cSH2 domain on the role of K15- dependent invasiveness (see above) and angiogenesis of KSHV infected endothelial cells [17]. As a first step, we performed an invasion assay using immortalized endothelial cells (HuAR2T) that had been transduced with a retroviral vector for K15P and a lentiviral vector for the PLCγ2 cSH2 domain. We observed that the presence of the PLCγ2 cSH2 domain significantly reduced K15-mediated invasiveness (Fig 8A and S3A Fig). We were able to confirm this result in the context of the HuAR2T cell line that had been stably infected with a recombinant KSHV carrying the K15P allele (HuAR2TrKSHV.219). In these cells, following the reactivation of the lytic cycle, the number of invading cells was significantly reduced in PLCγ2 cSH2-expressing cells (Fig 8B, compare lanes 1 and 3, and S3B Fig).

We have previously shown that K15 triggers angiogenesis *via* PLCγ1 and subsequent NFAT activation [17] and that the PLCγ2 cSH2 domain can reduce the level of NFAT activation in the case of the K15P allele (Fig 6B, 6C and 6E and Fig 7A). As a next step, we tested whether we could measure a reduction in K15-dependent angiogenic tube formation in primary endothelial cells (HUVEC) after the transduction with a lentivirus expressing the PLCγ2 cSH2 domain and a retroviral vector for K15P (Fig 8C). Indeed, in the presence of the PLCγ2 cSH2 domain, the increased formation of angiogenic tubes, which we previously showed to be induced by K15P [17], was significantly reduced (Fig 8C compare lanes 1 and 2, and S3C Fig). Remarkably, despite its inhibitory effect on K15-mediated angiogenesis, the PLCγ2 cSH2 domain did not decrease the number of capillary tubes in VEGF-stimulated HUVECs (compare lanes 5 and 6 of Fig 8C, and top row S3C Fig), thus indicating that the VEGF-dependent angiogenic signalling is not impaired by the PLCγ2 cSH2 domain. We further investigated the inhibitory effect of the PLCγ2 cSH2 domain on the increased angiogenesis displayed by KSHV infected primary endothelial cells, following the reactivation of the lytic cycle. To this end, we infected primary HUVECs with a recombinant KSHV (rKSHV.219) and, following lytic reactivation and transduction with the PLCγ2 cSH2 domain, we performed an *in vitro* capillary tube formation assay. As shown in Fig 8D and S3D Fig the angiogenic index was significantly increased in KSHV infected HUVECs, upon lytic reactivation (lane1 and 5), as compared to the uninduced samples (lanes 2 and 6) and to the uninfected controls (lanes 7–12). Transduction with the PLCγ2 cSH2 domain (lane 3), but not control vector (lane1), reduced the number of capillary tubes.

Therefore, we conclude that the isolated PLCγ2 cSH2 domain impaired both the increased invasiveness and angiogenesis observed in endothelial cells infected with a KSHV strain carrying the K15P allele.

## Discussion

Aberrant angiogenesis and cell migration represent important features in the pathogenesis of KS [49]. Our group previously showed that, in an *in vitro* assay (angiogenic tube formation on matrigel), performed in primary endothelial cells, KSHV triggers angiogenesis *via* activation of PLCγ1 by K15 [17]. In the present study, we demonstrate that K15 also triggers migration and invasiveness of KSHV infected endothelial cells in a PLCγ1-GIT1-βPIX-cdc42-dependent manner (Fig 1D and 1E). Confocal images showed that K15 co-localizes with PLCγ1 in KSHV infected endothelial cells (Fig 2A). K15 was found to partially co-localize with phosphorylated PLCγ1 (pTyr 783) (Fig 2B and 2C and Fig 3A–3C) and to co-localize strongly with βPIX (Fig 2B) and GIT1 (Fig 2C). In endothelial cell cultures, in which the lytic replication cycle had been activated, this co-localisation was mostly seen in orf59-negative cells, which are either latent or show a restricted viral gene expression pattern (Fig 3A–3C). Moreover, co-immunoprecipitation of PLCγ1 in virus infected endothelial cells showed the presence of a complex formed by K15, PLCγ1, GIT1, and cdc42 (Fig 3D and 3E). Similar results were obtained in the GST-pulldown assay performed with a GST-fused K15 cytoplasmic tail (Fig 6A). Since several studies reported previously that βPIX and GIT1 form a stable complex in endothelial cells [50,51], we hypothesize that K15 recruits a complex formed by PLCγ1, βPIX, GIT1 and cdc42, which is then responsible for the increased invasiveness of KSHV infected endothelial cells that are either latent or show a restricted viral gene expression pattern. In our invasion assay, the majority of migrating cells do not express RFP, a marker of early lytic gene expression in the recombinant KSHV.219 used in this study [52], suggesting that the majority of migrating cells have not entered the lytic cycle. On the other hand, we did have to activate the lytic cycle in order to observe an increased invasiveness of the KSHV infected HuAR2T cells. We therefore believe that additional viral or cellular factors, possibly acting in a paracrine manner, have to be released from the small percentage of lytic cells (see Fig 3A) in order to promote the invasiveness of the majority of KSHV infected cells that have not entered the lytic replication cycle. In this scenario K15 would therefore be a necessary (since its silencing abrogates invasiveness) but not sufficient factor for the increased invasiveness of KSHV infected endothelial cells.

PLCγ1 contains two SH2 domains (Fig 1A), the nSH2 domain, which binds to receptor tyrosine kinases (RTKs), and the cSH2 domain, which participates in an intra-molecular interaction necessary for PLCγ1 enzymatic autoregulation [47,48,53]. Similar to RTKs, both the M and P variants of K15 associate with the nSH2 domain of PLCγ1, since mutation of the conserved R586 in PLCγ1 responsible for contacting the phosphorylated tyrosine in the SH2 binding site of the interacting protein, decreases the affinity of both K15 variants for PLCγ1 (Fig 4). However, in the case of K15P, but not of K15M, mutation of a similar arginine (R694) in the PLCγ1 cSH2 domain reduced this interaction more significantly. Since the PLCγ1 cSH2 domain is involved in an intramolecular interaction with the phosphorylated Y783 (Fig 4A, [47,48,53]), one interpretation of our observation could be that a change in the PLCγ1 conformation, resulting from a loss of the intramolecular interaction between Y783 and the cSH2 domain, could dramatically affect the affinity for K15P. However, we also found that the cSH2 domain of PLCγ2 directly binds to the YEEV motif of K15P (but not K15M) (Figs 5A–5C, 7B and 7D). By analogy, we postulate that the PLCγ1 cSH2 domain can also directly interact with K15P. In view of the importance of the cSH2 domain for the enzymatic activation of PLCγ1, we speculate that the recruitment of this domain by K15P could explain the constitutive activation of PLCγ1 induced by K15P [17]. We previously reported that a mutation of either the tyrosine residue in the K15P YEEV motif or the proline residues in the K15P SH3 binding motif (PPLP), reduced the interaction with PLCγ1 and that both mutations together abolished it completely [17]. We therefore conclude that, most likely, the PLCγ1 SH3 domain also contributes to the recruitment of PLCγ1 by K15, in addition to either one or both the two SH2 domains.

Since our data suggest that the interaction between K15 and PLCγ1 contributes to both angiogenesis and invasiveness in KSHV infected endothelial cells, we wanted to explore the functional effects of disrupting this interaction. We have previously shown that the commercially available PLCγ1 inhibitor, U73122, blocks K15-dependent NFAT activation [17]. However, PLCγ inhibitors have so far not been adopted for clinical use. Therefore, to elucidate the contribution of the association between K15 and PLCγ1 to virus mediated invasiveness and angiogenesis, we wanted to target more specifically the K15-dependent activation of PLCγ1. Others had already shown, as evidence of an involvement of PLCγ1 in cell motility, that a dominant negative fragment of PLCγ1 can impair the migration and invasiveness of cancer cells *in vivo* [54]. Based on the observation that the isolated cSH2 domain of PLCγ2 interacts with the YEEV motif of K15P (Fig 5B) and that this interaction involves R672 of the PLCγ2 cSH2 domain (Fig 5C), we explored whether the PLCγ2 cSH2 domain could act as a dominant negative inhibitor of the K15P-PLCγ1 interaction. We showed that the PLCγ2 cSH2 domain can compete with PLCγ1, GIT1 and cdc42 for the binding to K15 (Fig 6A), releasing these factors from the complex and thereby decreasing the levels of PLCγ1 phosphorylation (Fig 6B) as well as the NFAT-dependent promoter activation (Fig 6C) induced by K15. Although the binding of K15 to PLCγ1 was only moderately impaired in the presence of the highest PLCγ2 cSH2 concentration (2 μg of transfected DNA) (Fig 6A, lane 4), a significant impairment in NFAT activation was observed already with lower amounts of the transfected PLCγ2 cSH2 domain (0.5 μg of transfected DNA) (Fig 6C). This result suggests that even a partial inhibition of the K15-PLCγ1 interaction, which as discussed above, likely involves also the K15 PPLP motif and the PLCγ1 SH3 domain, is sufficient to significantly decrease the K15-dependent intracellular signalling. In line with these data, the K15-dependent phosphorylation of PLCγ1 on Y783 in endothelial cells could be efficiently inhibited by overexpressing the PLCγ2 cSH2 domain (Fig 6B).

Activation of NFAT signalling by K15M, which binds PLCγ1 through its nSH2 domain (Fig 4B), and does not associate with the PLCγ2 cSH2 domain (Fig 7B and 7D), could not be significantly inhibited by overexpressing the PLCγ2 cSH2 domain (Fig 7A and 7C), thus suggesting that the inhibitory effect of the PLCγ2 cSH2 domain on K15P is due to its ability to interact with the latter, and not due to an indirect effect on other cellular proteins. Apart from binding to the phosphate moiety, which provides half of the ligand binding energy, SH2 domains also contact their cognate binding sites through additional residues in their binding pocket, which confer increased affinity and specificity [43]. We therefore hypothesize that different residues in the K15P and M SH2 binding sites, involved in contacting the phosphotyrosine-binding pocket allow K15P, but not M, to associate with the PLCγ2 cSH2 domain.

A recent report [46] showed that the affinity of SH2 domains for their ligands can be increased by altering amino acids which flank the binding pocket for the residues surrounding the phosphorylated tyrosine in the corresponding SH2 binding site. We therefore explored if the dominant negative effect of the PLCγ2 cSH2 domain on the K15P-PLCγ1 complex could be enhanced by strengthening its binding to K15P. We found (Figs 5D–5F, 6E and 6F) that altering two residues, S677V and R695L, achieves this objective and that the resulting PLCγ2 SB mutant (S677V/R695L) shows an increased ability to inhibit the K15-dependent activation of NFAT, but not of NF-κB, thus demonstrating the specificity for the K15-dependent recruitment and activation of NFAT. Using surface plasmon resonance we observed a similar affinity of the purified PLCγ2 cSH2 wt and SB domains for a purified GST-fusion protein containing the K15 cytoplasmic domain. On one hand, this experiment confirms that the PLCγ2 cSH2 wt and SB interact directly with the K15 cytoplasmic domain with significant affinity (K_D_ of 4–6 x 10^−9^). On the other hand, we only observed a moderate increase in affinity of the PLCγ2 cSH2 SB domain over the wt counterpart, unlike Kaneko et Al [46] who found a 300 fold increase in the affinity of the Fyn SH2 domain SB to the phosphorylated EGFR peptide. Since tyrosine phosphorylation (on the SH2 binding site) increases the affinity for the SH2 domain on the order of 100 fold [42,43], a possible interpretation of our results is that the K15 fusion protein used in our SPR assay was not phosphorylated and we therefore failed to observe an increased affinity of the SB domain when using recombinant proteins, in contrast to the results obtained when we analysed the binding of PLCγ2 cSH2 wt and SB domains in lysates of transfected cells (Figs 5E, 5F, 7B and 7D).

We could also show that the PLCγ2 cSH2 domain was able to impair the K15- and KSHV-mediated angiogenic effect (angiogenic tube formation) as well as the increased invasiveness of primary and immortalised endothelial cells (Fig 8 and S3 Fig). Together, the reported results demonstrate an involvement of the KSHV K15 protein in the increased migration, invasiveness and angiogenesis of KSHV infected endothelial cells. This function of K15 relies on the recruitment and activation of PLCγ1 and downstream signalling complex involving βPIX, GIT1 and cdc42. Therefore, at the molecular level, K15 exploits cellular mechanisms of integrin-dependent cell motility.

Our results, though, do not exclude the previously described involvement of other viral proteins [12,15,16,49,55] in KSHV-induced invasiveness and angiogenesis. In fact, the observation that K15 overexpression alone can induce invasiveness (Fig 1B and 1C) whereas in KSHV infected endothelial cells, the contribution of K15 the increased invasiveness only becomes measurable if there are lytic cells present in the culture (Fig 1D and 1E), suggests that, when expressed in its physiological context from the viral genome, K15 is necessary, but not sufficient, for KSHV-induced invasiveness and angiogenesis. We believe that the difference in invasiveness between K15 overexpression and its expression in the context of KSHV genome is due to a much broader range of cellular genes being induced by the former [17]. It is therefore plausible that, in KSHV infected cells, other viral proteins may complement the angiogenic effect of K15. This may include viral proteins typically expressed during the lytic replication cycle, which would explain why we observe increased invasiveness/angiogenesis (and a role for K15) primarily in cell cultures in which the lytic (productive) replication cycle has been induced (Figs 1D, 1E; and 8D). Taken together, our results indicate that the necessary role of K15 in KSHV-dependent angiogenesis/invasiveness could be a potential therapeutic target. We identified the isolated cSH2 domain of PLCγ2, as well as its SB derivative, as dominant negative inhibitors of K15P-dependent NFAT signalling and the resulting increased invasiveness and angiogenesis of endothelial cells infected with a KSHV strain carrying the K15P allele. This observation could provide the basis for developing small molecule inhibitors that would target the ability of KSHV to increase the invasiveness and angiogenesis of infected endothelial cells. Our findings therefore provide a proof of principle that the association between K15 and PLCγ1 represents a potential therapeutic target for the development of a protein-protein interaction (PPI) inhibitor able to counteract the increased invasiveness and angiogenesis of virus-infected cells. Although we have so far only been able to antagonize the recruitment of PLCγ1 by K15P, and not by its other allele, K15M, our results may provide the foundation for a search for dominant negative protein fragment-based inhibitors or small molecules which will also target K15M and help to mitigate some of the pathogenic features of KSHV in infected endothelial cells.

## Materials and Methods

### Ethics statement

The use of human umbilical cords was approved by the Hannover Medical School Ethics Committee and experiments were performed in agreement with the Declaration of Helsinki. Written informed consent was obtained from parents of umbilical cord donors.

### Plasmids

GST-K15 contains a synthetic K15P cytoplasmic tail (aa 347–489)(sK15P), with a nucleotide sequence optimized for prokaryotic expression (GENEART GmbH). The sK15P fragment was inserted into pGEX-6P-1 using EcoRI and BamHI sites.

The K15-P and M wt constructs were previously described [22,25,56]. The NF-κB reporter p3EnhκB conA–Luc construct was kindly provided by A. Eliopoulus (University of Crete Medical School, Heraklion, Greece). The reporter vector pNFAT-TA-Luc, which contains three NFAT binding sites cloned from IL2 promoter upstream of the firefly luciferase gene, and the corresponding control vector pTA-Luc were purchased from Clontech. Vectors expressing PLCγ1wt and PLCγ2 specific array domains (cSH2, nSH2, SH3, spPH) were kindly provided by M. Katan (University College London, London, UK); all of these were based on the pTrieX-4 vector [53]. PLCγ1 R586L and PLCγ1 R694L were generated by site-directed mutagenesis of PLCγ1 wt using the following primers: F 5′-CTTCCTCGTGCTAGAGAGTGA-3′; R 5′-CTCACTCTCTAGCACGAGGAA-3′; F 5′-CCTTCCTGGTGCTGAAGCGGAATGAACCC-3′; R5′-GGGTTCCTTCCGCTTCAGCACCAGGAAGG-3′, respectively. PLCγ2 cSH2 R672L was generated by site-directed mutagenesis of PLCγ2 cSH2 using the primers F 5′-CTTCCTGATCCTGAAGCGAGAGGG-3′ and R 5′- CCCTCTCGCTTCAGGATCAGGAAG-3′. PLCγ2 cSH2 S677V was generated by site directed mutagenesis of PLCγ2 cSH2 wt using the primers:F 5`-CATAGGAGTCGACCCCCTCTC-3`and R5`-GACAGGGGGTCGACTCCTATG-3`. PLCγ2 cSH2 R695L was generated by site directed mutagenesis of PLCγ2 cSH2 wt using the primers:F5`-AAAGCATTGTCTCATCAACCG-3`; and R5`-CGGTTGATGAGACAATGCTTT-3`. To generate the PLCγ2 cSH2 S677V/R695L (SB) mutant, the S677V mutant was amplified using the R695L primers. In order to generate the pRRL.PPT.SF.PLCγ2 cSH2 lentiviral construct, the DNA fragment containing PLCγ2cSH2 fused with the pTriEx-4 containing tags (S-tag, and His-tag) was amplified using the primers F5′-CCTGCTAGCTCGGACCGAAATTAATACG3′and R5′-GGTACATGTTTACGTTGAGGAGAAGCCCGG-3′. The amplified segment was then inserted in the lentiviral pRRL.PPT.SF.GFP vector (kindly provided by A. Schambach, Hannover Medical School) using the NheI and BsrgI sites.

### Cells, transfections, infection and virus production

All cells were cultured at standard conditions: 37°C in a 5% CO_2_ incubator. HEK293T (ACC 305 from the German Collection of Microorganisms and Cell Cultures-DMSZ) HEK293 (ATCC CRL-1573) and HeLa CNX (ACC 57 from the German Collection of Microorganisms and Cell Cultures-DMSZ) cells were cultured in Dulbecco Modified Eagle′s Medium (DMEM) (Cytogen) supplemented with 10% foetal calf serum (Hyclone). HEK293 stably infected with KSHV BAC36 wt or ΔK15 have been previously described [17]. Human umbilical vein endothelial cells (HUVECs) were freshly isolated from umbilical cords by collagenase digestion as described previously [57] and grown in EGM-2MV medium (Lonza). HuAR2T, an endothelial cell line obtained from HUVECs conditionally immortalized with a doxycycline inducible human telomerase reverse transcriptase (hTERT) and simian virus 40 (SV40) large T antigen transgene expression [58], (a kind gift of Dagmar Wirth, HZI, Braunschweig) was grown in EGM-2MV medium containing 200 ng/ml of doxycycline. The HuAR2T cell line stably harbouring recombinant KSHV.219 (r.KSHV.219) was obtained as previously reported [59]. For transfection of HeLa CNX and HEK293T, either 1-2x 10^5^cells/ml were plated in six well plates, twenty-four hours later FuGENE transfection reagent (Promega) was used at a ratio 3μl:1μg of DNA. 1μg of each construct was transfected, unless otherwise stated. For transfection of small interfering RNA (siRNA) into HUVECs and HuAR2T, 100pmole of siRNA were transfected into 10^5^ cells using the Neon transfection system (Invitrogen) according to the manufacturer′s instructions. The following siRNAs (siGenome SMART pool) were purchased from Dharmacon, Thermo Scientific: control (non-targeting siRNA pool 2 D-001206-14-20), siPLCγ1 (M-003559-01), siβPIX (M-009616-00), siGIT1 (M-020565-02) and siRNA against KSHV K15 protein targeting exon 8 (CAACCACCUUGGCAAUAAU) [17,22,25]. For retrovirus production, HEK293T were transfected using the calcium phosphate method with either pSF91-K15-IRES or pSF91-IRES vector [17], together with the packaging plasmids pM57DAW (gag/pol) and pRD114 envelope protein. Retroviruses containing pSF91-IRES or pSF91-IRES-K15 were produced as described previously [17]. For lentivirus production, HEK293T were transiently co-transfected using the calcium phosphate method with the helper plasmids (pMDLGg/p, pRSV-REV and pMD.G) and either pRRL.PPT.SF.GFP or the pRRL.PPT.SF.PLCγ2 cSH2 plasmid; lentiviral stocks were produced as described previously [59]. For transduction, cells were transduced with the indicated lenti- or retrovirus in the presence of 5 μg/ml of polybrene and centrifuged for 30 min at 450g. Medium was changed 8 hours post transduction. Experiments were performed with 60–70% of transduced cells, 48 hours post transduction. Sf9 insect cells (ACC 125 from the German Collection of Microorganisms and Cell Cultures-DMSZ) were grown in Grace′s medium (Gibco) supplemented with 10% fetal bovine serum, 100 U/ml of penicillin and 50 ug/ml of streptomycin. Cloning and production of RTA expressing baculovirus were previously described [52].

### Production of recombinant KSHV virus

rKSHV.219 was produced from BJAB (ACC 757 from the German Collection of Microorganisms and Cell Cultures-DMSZ) infected with rKSHV.219 as described in [60]. Briefly, 6x10^5^ BJAB rKSHV.219 were grown for seventy-two hours in spinner flasks (agitation at 60rpm) in RPMI1640 medium supplemented with 10% FCS and in the presence of 2.5 μg/ml of anti-IgM antibody. Subsequently, the culture was centrifuged and the supernatant was first filtered (0.45 μm pore size filter) and then centrifuged 5 hours at 15000 rpm. The pellet was resuspended in EBM2 medium.

### Production and purification of GST- and His-tag fusion proteins

A codon optimized version for bacterial usage of the cytoplasmic tail of K15P (aa 347–489) was purchased from GENEART and cloned in pGEX-6P using BamHI and EcoRI restriction sites. The plasmid was transformed in Rosetta *E*. *coli* and grown at 37°C in LB medium with ampicillin. Once an optical density at 600nm (OD600) of 0.8 was reached, bacterial cultures were induced for 4 hrs with 1mM isopropyl-γ-D-thiogalactopyranoside (IPTG) at 30°C and subsequently pelleted by centrifugation. Bacteria were lysed in 1x PBS plus protease inhibitors and sonicated 3 times for 30 seconds. Subsequently, 0.1% Triton-X 100 was added and the lysate was cleared by centrifugation at 14000 rpm for 10 minutes. Cleared lysate was incubated with glutathione sepharose beads (Amersham Bioscience) overnight at 4°C. The PLCγ1 and 2 cSH2 domains were produced and purified as previously described [53]. Briefly, plasmids harbouring the PLCγ1 or 2 cSH2 domains were transformed into Rosetta *E*.*coli* and grown to an OD600 of 0.4. Protein expression was induced with 100 μM IPTG for approximately 10 hours at 25°C. Bacteria were pelleted and stored at -20°C until further processing. Pellets were resuspended in 10 ml of chilled lysis buffer (25mM tris HCl, 250mM NaCl, 40mM imidazole, 10mM benzamidine, 1mM MgCl_2_ and 100μM CaCl_2_) per 1L of culture. Lysis was further accomplished by sonication (3x30 sec) and by the subsequent addition of 10% of Triton X-100. Bacterial lysate was cleared by two rounds of 15 min centrifugation at 14000g at 4°C. Supernatant was then incubated with 2 ml washed Ni beads (Quiagen), for 4 hours rolling at 4°C. Subsequently beads were washed 3 times with lysis buffer and stored at -80°C.

### Production of K15 monoclonal antibody

50 μg of the purified GST-K15 (aa 347–489) were injected intraperitoneally (i.p.) and subcutaneously (s.c.) into LOU/C rats using incomplete Freund's adjuvant supplemented with 5 nmol CpG 2006 (TIB MOLBIOL, Berlin, Germany). After six weeks interval a final boost with 50μg K15 and CpG 2006 was given i.p. and s.c. three days before fusion. Fusion of the myeloma cell line P3X63-Ag8.653 with the rat immune spleen cells was performed according to standard procedures. Hybridoma supernatants were tested in a solid-phase immunoassay with K15-GST or GST bound to ELISA plates *via* mouse anti-GST antibody. Antibodies from tissue culture supernatant recognizing K15 were detected with HRP conjugated mouse mAbs against the rat IgG isotypes (TIB173 IgG2a, TIB174 IgG2b, TIB170 IgG1 all from ATCC, R-2c IgG2c homemade), thus avoiding mAbs of IgM class. HRP was visualized with ready to use TMB (1-Step Ultra TMB-ELISA, Thermo). Monoclonal antibodies (mAbs) that reacted specifically with the K15 were further analysed by western blot and IFA. In experiments, antibodies from clone number 10A6 (IgG G1) and 18E5 (IgG 2b) were used for western blot and immunofluorescence, respectively.

### Western blot analysis and antibodies

In order to assay the expression levels of specific proteins, cells were lysed in 1x SDS sample buffer (62.5 mM tris-HCl pH 6.8, 2% w/v) SDS, 10% glycerol, 50 mM DTT, 0.01% (v/v) β-mercaptoethanol and 0.01% (w/v) bromophenol blue). For the detection of K15 protein, samples were not boiled prior to SDS-PAGE. Proteins were separated by SDS-PAGE and transferred to nitrocellulose membrane (Amersham). Membranes were blocked 1 hour in 5% (w/v) milk in PBS-T. Specific proteins were identified using the following primary antibodies: rat monoclonal anti-K15 antibody (see above), rabbit anti-PLCγ1(#2822), rabbit anti-phospho Y783 PLCγ1(#,2821), rabbit anti S tag (#8476), rabbit anti-cdc42 (#2462) were purchased from Cell Signaling Technology; mouse anti-KSHV ORF 45 (sc-53883), mouse anti-KSHV Kb-Zip (sc-69797), mouse anti-βPIX (sc-136035) were obtained from Santa Cruz Biotechnolocy Inc.; rat anti KSHV ORF73 (LNA-1)(13-210-100) and mouse anti-β-actin (A5441) were purchased from Advanced Biotechnologies, and Sigma Aldrich, respectively; mouse anti GIT (611388) was obtained from BD Transduction Laboratories; mouse anti KSHV ORF 59 was purchased from Advanced Biotechnology Inc. (13-211-100). A polyclonal rabbit antibody to the FLAG epitope was purchased from Sigma (F7425). The HRP-labelled polyclonal secondary antibodies: goat anti rabbit (P0448), rabbit anti mouse (P0260) and rabbit anti rat (P0450) were purchased from DAKO.

### *In vitro* invasion assay

Matrigel-coated invasion chambers (354483, BD Biosciences) were used. HuAR2T or HuAR2Tr.KSHV were transfected with the indicated siRNA, twelve hours later cells were transduced with the indicated lenti- or retroviral vectors. Twenty-four hours after siRNA transfection, the lytic cycle was induced with 10% or 15% RTA (v/v) supernatant and 1mM sodium butyrate; twenty-four hours later, cells were starved in EBM2 supplemented with 2% FBS for twelve hours and subsequently cells were seeded in quadruplicate in Matrigel-coated invasion chambers under starvation conditions and were allowed to invade for twenty-four hours.

Alternatively, uninfected HEK293 cells, or HEK293 cells stably harbouring KSHV BAC36 wt or a ΔK15 mutant, were plated on a 6-well plate at a density of 6X10^5^ cells per well and the lytic cycle was induced twenty-four hours later. Cells were starved overnight in DMEM and thirty-six hours after induction, 5x10^4^ cells were plated in 0.5ml of DMEM with 0.1% BSA in quadruplicate in the inner insert of an invasion chamber. In the outer insert 0.75ml of DMEM supplemented with 5% FCS was added and the cells were incubated for twenty-four hours. Before plating of cells on the inner chamber, the Matrigel inserts were rehydrated with 0.5 ml of DMEM for two hours. Subsequently, cells were washed in 1xPBS, fixed in 4% PFA, permeabilized with 0.2% Triton X-100, stained with DAPI (Sigma-Aldrich) and the bottom of the chambers was cut out and mounted on coverslip,. Invading cells were counted using a fluorescence microscope. Cells were counted in four random fields per chamber using Cell Profiler 2.0 software [61]. Each experiment was performed three times. To determine whether there was a statistically significant difference between the different conditions, a Kruskall-Wallis test with a Dunn’s post- test was performed.

### Immunofluorescence assay

2.5x10^4^ HuAR2T r.KSHV.219 cells were plated on glass coverslips and thirty-six hours later washed with 1xPBS and fixed with 100% ice cold methanol for 20 min at -20°C. Cells were then thoroughly washed with 1xPBS and incubated for one hour in 10% FCS in PBS at 37°C. Coverslips were then incubated with a rat monoclonal antibody to K15 (clone 18E5) for one hour at 37°C, washed 3 times in 1x PBS and incubated for one hour with Cy3-labelled anti-rat secondary antibody (712-165-153 Jackson Immuno Reasearch) for one hour at 37°C. Coverslips were washed, fixed with 4% PFA 20 minutes, PFA was quenched with 1xTBS 10 min at room temperature. Coverslips were then stained as indicated. The following fluorescently labelled secondary antibodies were used: FITC conjugated donkey anti-rabbit (711-095-152 Jackson Immuno Research), Cy5 conjugated goat anti-mouse (115-175-164 Jackson Immuno Research). Images were taken with ZEISS LSM 510 Meta scan head connected to an inverted microscope Axiovert 200M. To analyse the co-localisation the JACOP tool was used and the Pearson’s coefficient (PC) was calculated and shown in Figs 2 and 3A[52].

### Co-immunoprecipitation and pull-down assays

Thirty hours after transient transfection with the indicated plasmid, HEK293T were lysed in IP buffer (150mM NaCl, 25mM tris HCl pH7,6, 1mM EDTA, 1% glycerol, 0.2% NP-40). 200 μl of precleared lysate was incubated overnight with gentle shaking at 4°C with appropriate beads, as indicated in the figure legends. For the S-tag constructs S protein agarose beads (Novagen) were used. For PLCγ1 immunoprecipitation, protein A sepharose beads (GE Healthcare) were incubated with pre-cleared HuAR2T r.KSHV.219 lysate and anti-PLCγ1 antibody according to the manufacturer′s instructions. The His-tag pull-down assay was performed by incubating overnight, with gentle shaking at 4°C, equal amounts of Ni beads (Quiagen) bound with the indicated fusion proteins together with pre-cleared lysate of HEK293T cells. GST pulldown assays were performed by incubating equal amounts of GST-fusion protein and control GST coated beads with pre-cleared cellular lysate of HEK293T cells transfected with the indicated constructs overnight with gentle shaking at 4°C. Beads were washed three times in ice cold lysis buffer and the bound proteins complexes were analysed by SDS-PAGE and western blot.

### Luciferase-based reporter assay

HEK293T or HeLa cells were transiently transfected in six well plates with 50 ng of reporter plasmid and the indicated expression constructs. Forty hours after transfection cells were harvested in 300 μl/well of 1x reporter lysis buffer (Promega). Luciferase activity was measured in cleared lysates with a luciferase buffer according to the manufacturer’s instructions (Promega). Each experiment was performed at least three times in duplicate; the error bars in the graphs represent the standard deviation across all three experiments.

### Determination of binding affinity constants by surface plasmon resonance

Surface plasmon resonance (SPR) was performed using a Biacore X100 (GE Healthcare) instrument. Purified recombinant GST-K15P cytoplasmic tail, dissolved in acetate buffer at pH 5.5, was immobilized in the Flow cell 2 of a CM4 chip by amine-coupling until reaching 207 relative units (*Rmax* approx. 100 RU). For binding assays, purified recombinant His-tagged wild type and mutant PLCγ2 cSH2 domains were injected at different concentrations in HBS-EP buffer (10 mM Hepes, 150 mM, NaCl, 3 mM EDTA, 0.005% (vol/vol) surfactant P20, pH 7.4) at a flow rate of 10 μl/min. To determine the association and dissociation constants single cycle kinetics analysis was used. Briefly, 1.5, 3, 6, 12, 24 nM of wild type and SB PLCγ2 cSH2 domain were injected at a flow rate of 30 μl/min in HBS-EP buffer during 120 sec followed by 900 sec of dissociation. The chip surface was regenerated by injecting 2M MgCl_2_. The sensorgrams obtained were analysed with the Biacore X100 Evaluation 2.0.1 software. Bulk refractive index changes were removed by subtracting the reference flow cell (Flow cell 1) responses, and the average response of a blank injection was subtracted from all analyte sensorgrams to remove systematic artefacts.

### *In vitro* capillary tube formation assay

HUVECs were either transduced with the indicated retroviral vectors or infected (MOI 20) with rKSHV.219. In the case of transduced cells, they were starved in EBM2 medium supplemented with 2% FCS for thirty hours after transduction. Forty-eight hours after transduction the assay was performed. Three days after infection with recombinant KSHV, HUVECs were transduced, with the lentiviral vector carrying either the PLCγ2 cSH2 domain or the control vector. Four days after infection, infected HUVECs were treated with 1mM of sodium butyrate and 10–15% RTA to induce the lytic cycle. The capillary tube formation assay was performed thirty-six hours after reactivation. For this assay, 8x10^3^ cells were plated in wells precoated with growth factor reduced matrigel (BD Biosciences) and incubated in a 37°C, 5% CO2 incubator according to the manufacturer′s instructions and as previously reported [17]. For each well four different fields were photographed with a NIKON T200 fluorescence microscope. The angiogenic index was calculated as the number of branching points in a visual field after 4–6 hours of incubation. The number of branching points was averaged and standard deviation ±95% CI was calculated. Each experiment was performed independently three times in quadruplicates.

To determine whether there was a statistically significant difference between the different conditions, a Kruskall-Wallis test with a Dunn’s post- test was performed.

## Supporting Information

S1 Fig Representative images of invasion assay. (A) Images of invading HuAR2T transduced with a K15-expressing retrovirus. For quantification, see Fig 1B; (B) Images of invading HuAR2T transduced with a K15-expressing retrovirus and silencing of PLCγ1. For quantification, see Fig 1C; (C) Images of invading HuAR2TrKSHV.219 after induction of the lytic cycle, following silencing of PLCγ1 or K15. For quantification, see Fig 1D; (D) Images of invading HuAR2TrKSHV.219 after induction of the lytic cycle, following silencing of GIT1, βPIX or cdc42. For quantification, see Fig 1E.
(EPS)

S2 Fig Deletion of K15 from the KSHV genome reduces the invasiveness of infected cells. (A) Invasion assay using uninfected HEK293 cells (-), or HEK293 cells stably infected with KSHV BAC36 wt or KSHVΔK15 mutant, following induction of the lytic cycle. (B) Representative images of invading cells.
(EPS)

S3 Fig Representative images of invasion and capillary tube formation assay. A) Images of invading HuAR2T transduced with a K15-expressing retrovirus and a lentivirus for the PLCγ2 cSH2 isolated domain (pRRL.PT.SF.PLCγ2 cSH2) or the control lentivirus (pRRL.PT.SF). For quantification, see Fig 8A and 8B) Images of invading HuAR2TrKSHV.219 after induction of the lytic cycle and transduction with a lentivirus for the PLCγ2 cSH2 domain (pRRL.PT.SF.PLCγ2 cSH2) or the control lentivirus (pRRL.PT.SF). For quantification, see Fig 8B and 8C) Images of angiogenic tubes formed in HUVECs following transduction with the PLCγ2 cSH2 domain or control vector (pRRL.PT.SF) and treatment with VEGF (top row), transduction with K15 (middle row) or the control vector for K15 (pSF91). For quantification see Fig 8C and 8D) Images of angiogenic tubes formed by KSHV infected HUVECs following the induction of lytic reactivation (top row) or left uninduced (bottom row) and transduced with the indicated lentiviral vectors or left untransduced. For quantification, see Fig 8D.
(EPS)
